# Increased frequency of FBN1 frameshift and nonsense mutations in Marfan syndrome patients with aortic dissection

**DOI:** 10.1002/mgg3.1041

**Published:** 2019-12-12

**Authors:** Shijun Xu, Lei Li, Yuwei Fu, Xin Wang, Hairui Sun, Jianbin Wang, Lu Han, Zining Wu, Yongmin Liu, Junming Zhu, Lizhong Sun, Feng Lan, Yihua He, Hongjia Zhang

**Affiliations:** ^1^ Department of Cardiac Surgery Beijing Anzhen Hospital Capital Medical University Beijing China; ^2^ Beijing Institute of Heart, Lung and Blood Vessel Diseases Beijing China; ^3^ Beijing Lab for Cardiovascular Precision Medicine Beijing China; ^4^ Beijing Aortic Disease Center Cardiovascular Surgery Center Beijing China; ^5^ Beijing Engineering Research Center for Vascular Prostheses Beijing China; ^6^ Department of Echocardiography Beijing Anzhen Hospital Capital Medical University Beijing China

**Keywords:** aortic aneurysm, aortic dissection, FBN1, Marfan syndrome

## Abstract

**Background:**

Marfan syndrome (MFS) is an inherited connective tissue disease that mainly involves Fibrillin‐1 (FBN1) mutations and aortic manifestations. In this study, we investigated the correlations between the FBN1 genotype–phenotype and aortic events (aortic dissection and aortic aneurysm) in patients with Marfan syndrome.

**Methods:**

Genotype and phenotype information was evaluated in 180 patients with MFS. DNA sequencing was performed on each patient. According to the clinical manifestation, these patients were split into two groups: the aortic dissection group and the aortic aneurysm group. Aortic wall tissue was obtained from Marfan patients who underwent surgery and was used for staining.

**Results:**

A total of 180 patients with FBN1 mutations were grouped into four categories: 90 with missense mutations, 32 with splicing mutations, 29 with frameshift mutations, and 29 with nonsense mutations. There was a significantly higher frequency of frameshift and nonsense mutations observed in aortic dissection than in aortic aneurysm (25.58% vs. 4.35%, *p* = .005; 25.58% vs. 8.70%, *p* = .033, respectively;), while missense mutations showed a higher frequency in aortic aneurysm than in aortic dissection (69.57% vs. 32.56%, respectively; *p* < .001) and a higher rate of lens dislocation (34.78% vs. 13.95%, respectively; *p* = .008). Pathological staining showed that elastic fibers were sparser in patients with a frameshift and nonsense mutations, and the smooth muscle cells were sparser and more disorganized than those observed in patients with missense mutations.

**Conclusion:**

This study showed that FBN1 gene frameshift and nonsense mutations are more common in patients with aortic dissection and may have meaningful guidance for the treatment of Marfan syndrome patients.

## Graphical abstract

This study increase our understanding of the FBN1 gene mutation spectrum in Chinese patients with MFS. We found that frameshift mutations were more common in patients with aortic dissection and that the aortic wall structure contained fewer elastic fibers and fewer and more disordered smooth muscle cells.

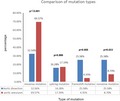

## 1 INTRODUCTION

Marfan syndrome (MFS) (OMIM #000138) is an autosomal dominant hereditary connective tissue disease typically involving the Fibrillin‐1 (FBN1) mutations. MFS mainly affects cardiovascular, ocular, and skeletal systems and less frequently involves lung, muscle, skin, and adipose tissues (Dietz et al., 1991). The effects of MFS on the cardiovascular system, especially aortic dissection (AD) and aortic aneurysm (AA), are the main cause of death (Dietz et al., 1991). However, AD is more dangerous because of the high rates of sudden death and operative mortality, which can reach 23.9% (Rampoldi et al., 2007). Although AA is also an important cause of death in MFS, the operative mortality is only approximately 2% (Mookhoek et al., 2016). Therefore, determining the risk factors of AD in patients with MFS would be especially important and meaningful for the treatment of these patients.

At present, MFS treatment follows the 2014 ESC guidelines (Mookhoek et al., 2016). Surgery is performed on patients with MFS who have a maximal aortic diameter ≥ 50 mm. A lower threshold of 45 mm is considered for patients with additional risk factors, including a family history of dissection, a size increase of 0.3 mm/year, severe aortic regurgitation, or a desire for pregnancy. However, according to our observations, a large number of patients with MFS have AD when the aortic root diameter is less than 50 mm; therefore, these patients need to be identified to assess the risk of AD. The MFS diagnosis is currently based on the revised Gent criteria (Loeys et al., 2010); compared with the previous standard (Rose et al., 2000), the Gent criteria place a stronger emphasis on genetic sequencing. Moreover, genetic sequencing (Podnar, Deiderick, Huerta, & Hunicke‐Smith, 2014) is becoming simpler and more practical to perform and allows MFS patients to be identified early, which is especially important in asymptomatic parents.

Currently, over 2,900 distinct FBN1 mutations have been identified in patients with MFS (Franken et al., 2017). These mutations are unique to a family or individual. Missense mutations are the major type found in patients with MFS, and these mutations mainly affect cysteine residues (Franken et al., 2017). Many studies have explored the correlations between gene mutation types and clinical phenotypes in patients with MFS (Rommel et al., 2005; Stheneur et al., 2014). However, no specific links have been discovered with the exception of neonatal Marfan syndrome. These patients have severe symptoms and often die very early, with mutations occurring between 24 and 32 exons. Franken et al. (Franken et al., 2017) found that patients with a haploinsufficiency (HI) mutation have a higher probability of aortic events (AD and prophylactic aortic surgery). Linnea M et al. (Franken et al., 2017) found that the ratio of FBN1 truncation mutations was also higher in the aortic events in patients with MFS. These patients must be strictly supervised, but these findings are not sufficient to provide meaningful guidance for clinicians to treat MFS patients. Moreover, such studies have not been extensively performed in Chinese patients. Therefore, the main purpose of this study was to define the FBN1 gene mutation epidemiology in a large cohort of Chinese patients with MFS and to investigate the correlations between FBN1 gene mutation types and clinical phenotypes, especially with AD and AA.

## 2 MATERIALS AND METHODS

### 2.1 Study population

In this study, patients diagnosed with or suspected of having MFS were referred from the Department of Cardiovascular Surgery Center in Beijing Anzhen Hospital from January 2017 to December 2018. During this period, a total of 180 patients with FBN1 pathogenic or likely pathogenic variants were included in this analysis. The study was approved by the Editorial Policies and Ethical Considerations committee of this hospital. All subjects provided informed consent (for subjects who were younger than 18, consent was signed by their parents), and all information relating to individuals participating in the study was kept strictly confidential. The treatment of MFS patients followed the 2014 ESC guidelines. Each patient underwent rigorous imaging follow‐up (echocardiography or thoracic‐abdominal aorta computed tomography angiography examination) with a follow‐up interval of 1 year.

### 2.2 Data collection

Detailed clinical data were collected from these patients and included age, sex, suspected diagnosis, a medical history, family history, a physical examination, and routine laboratory test results. In addition, depending on the need for MFS diagnosis, echocardiography or thoracic‐abdominal aorta computed tomography angiography examination was performed to evaluate the condition of the heart and aorta. The clinical information covered all items in the diagnostic criteria for MFS. Thoracic deformity was defined as pectus carinatum deformity, pectus excavatum, or chest asymmetry. Lung disease was defined as pneumothorax. Skeletal deformity was defined as hindfoot deformity, plain pes planus, or protrusio acetabuli or a reduced upper segment (US)/lower segment (LS) ratio and an increased arm/height ratio or reduced elbow extension. To ensure the objectivity and accuracy of the information, information collection was performed by two different doctors, and any controversial information was finally determined by a third doctor. Venous blood (4 ml) was collected from all included individuals and stored in an ethylenediaminetetraacetic acid–anticoagulated tube as whole blood. Based on the previous literature and our previous research results, we identified genes related to aortic diseases and designed a hereditary aortic disease gene panel (Guo et al., 2017). All samples were subjected to panel sequencing. Aortic wall tissue was obtained from MFS patients who underwent surgery on the premise that the collection did not affect the therapeutic effect. The correlations between genotypes and clinical phenotypes were further explored by comparing the aortic wall tissue histological specimens of each genotyped patient. In addition, the pathological aortic specimens associated with different mutation types were stained to observe the effect of different mutation types on aortic wall structure. Peripheral blood separation was performed as follows: Fresh peripheral blood was placed in a refrigerated centrifuge and spun at 4°C at 1,600 *g* for 10 min. The low‐speed centrifugation aspirate containing white blood cells was removed, placed in a 200‐µL cryotube and stored at −80°C.

### 2.3 Preparation of DNA libraries

Genomic DNA was broken using a Covaris sonifier to prepare a DNA library. Probe sequences were designed for genes associated with aortic disease. Liquid‐phase hybridization was performed using biotin‐labeled probes and a library with specific index pooling. The probe coverage area was the target area for sequencing. No new genes were added to the panel during the study.

### 2.4 Bioinformatics analysis and interpretation of mutations

Quality control of raw reads was carried out for each sample. A Perl script was used to remove the connector sequences from the reads, and then, the read was pruned and filtered based on the read quality information**.** The read sequence was compared with the human genome by alignment software, and the alignment result (a bam file) was obtained. The nonspecific alignment sequence in the bam file was removed, and the realigned tool in the GATK software package was then used to rematch the bam file to improve the accuracy of the comparison near indels.

The final bam file and the location file for the target area were entered into the Unified Genotype tool of GATK, and the parameters were adjusted to obtain the sequencing depth and genotype information for each location in the target area. A mutation site (single nucleotide polymorphism (SNP) site) in the target region was obtained, and the SNP site was annotated with ANNOVAR to obtain information regarding the mutation, including disease‐related information, from a known database.

Standard genetic information analysis procedures were used to detect SNPs and small indels (indel), and the mutation sites were detected, filtered, and annotated. DGV (http://dgv.tacg.ca/gdv/app/home), OMIM (http://www.ncbi.nlm.nih.gov/omim), and Decipher (https://decipher.sanger.acdatabases such as.uk/) were used to comprehensively analyze and label the detected mutations. Software such as Provean and SIFT were used to predict the hazards of the variant genes to determine their pathogenicity. If the mutation was pathogenic/potentially pathogenic, the defined gene sequencing result was considered positive, whereas if the mutation was benign/possibly benign/clinically unclear, the defined gene sequencing result was considered negative. For results that met the Ghent diagnostic criteria but had negative sequencing results, full‐exome sequencing and multiplex ligation probe amplification (MLPA) were performed. Sanger sequencing was finally performed on the SNP/indel family.

### 2.5 Classification of alterations

The American College of Medical Genetics and Genomics (ACMG) variant classification recommendations were utilized for all reported variants (Richards et al., 2015). Notably, in missense variants, meeting the following two conditions (Loeys et al., 2010) was considered strong evidence of pathogenicity: “Well‐established in vitro or in vivo functional studies supportive of a damaging effect on the gene or gene product,” according to the ACMG guidelines:

- A missense variant that created or destroyed a cysteine residue;
- A missense variant that affected conserved residues in the EGF‐like domain consensus sequence (D/N) X (D/N) (E/Q) Xm (D/N) Xn (Y/F) (m and n represent variable numbers of residues).

**Positive result**: pathogenic or likely pathogenic variant(s) in a known disease gene associated with the reported phenotype.

**Possible diagnosis**: variant(s) in a known disease gene possibly associated with the reported phenotype. This category includes novel variants, including missense variants or in‐frame insertions/deletions in disease genes, that overlap the phenotype provided for the patients and a single rare or novel highly suspicious variants of uncertain significance (VUS) known to be in trans with a pathogenic/likely pathogenic variant in a gene that explains the reported phenotype.

**Candidate gene**: variant(s) predicted to be deleterious in a novel candidate gene that has not previously been implicated in MFS or for which the published data supporting an association may not yet be definitive. Supporting data could be based on model organism data, copy number variant data, tolerance of the gene to sequence variation, data regarding tissue or developmental timing of expression, or knowledge of the gene function and pathway analysis. Further research is required to evaluate any of the suggested candidate genes.

**Uncertain result:** VUS in a known disease gene and a patient phenotype consistent with the reported disease spectrum (e.g., uncertainty is limited to the pathogenicity of the variant due to a lack of parent samples to assess for de novo occurrence and determining the phase of variants in recessive disorders). This category also includes recessive conditions that overlap with the phenotype provided for the fetus in which only a single pathogenic/likely pathogenic variant is identified.

**Negative result**: no variants in genes associated with the reported phenotype identified.

### 2.6 Statistical analysis

The quantitative data are presented as the mean ± standard deviation. Qualitative variables are presented as frequencies and percentages. Quantitative variables were compared using Student's *t* test. Qualitative variables were compared using the x^2^ test or Fisher's exact test when appropriate. A value of *p* ≤ .05 was considered significant. Statistical analysis was performed with Empower Stats and verified by SPSS 20.0, and the results were found to be consistent.

## 3 RESULTS

### 3.1 Characteristics of patients with Marfan syndrome

In all, 180 patients were included in the analysis. The median age at diagnosis was 26.0 years (interquartile range, 11.25–32.75). A total of 97 patients (53.89%) were male. A large number of patients demonstrated aortic disease involving AD (43 patients, 23.89%) and AA (46 patients, 25.56%). There were 45 patients (25.00%) with lens dislocation and 18 patients (10.00%) with mitral valve prolapse. There were 90 missense mutations (50.00%), 32 splicing mutations (17.78%), 29 frameshift mutations (16.11%), and 29 nonsense mutations (16.11%). The detailed data are shown in Table 1. The clinical data and FBN1 mutations at gene and protein level for each patient, including information on familiarity and other vessel or cardiology features involvement, are shown in Table S1.

**Table 1:** Characteristics of Marfan syndrome patients with FBN1 mutation

Variables	All patients (*n* = 180)
Basic features	
Age of onset (year)	26.00 (11.25–32.75)
Body mass index (kg/m^2^)	19.98 ± 4.36
Gender (male)	97 (53.89%)
Family history	107 (59.44%)
System score	5.00 (3.00–7.00)
Cardiovascular features	
Sinus diameter (mm)	43.94 ± 15.50
Ascending aorta diameter (mm)	43.64 ± 16.37
Descending aorta diameter (mm)	28.55 ± 4.41
Hypertension	6 (3.33%)
Aortic aneurysm	46 (25.56%)
Aortic dissection	43 (23.89%)
Aortic insufficiency	
Mild	31 (17.22%)
Moderate	3 (1.67%)
Severe	23 (12.78%)
Mitral regurgitation	
Mild	37 (20.56%)
Moderate	3 (1.67%)
Severe	6 (3.33%)
Mitral valve prolapse	18 (10.00%)
Ocular features	
Lens dislocation	45 (25.00%)
Myopia	106 (58.89%)
Skeletal features	
Skeletal deformity	123 (68.33%)
Wrist sign	113 (62.78%)
Finger sign	111 (61.67%)
Wrist and finger sign	113 (62.78%)
Thoracic deformity	70 (38.89%)
Scoliosis	33 (18.33%)
Lung	14 (7.78%)
Hernia	12 (6.67%)
Type of mutation	
Missense mutation	90 (50.00%)
Splicing mutation	32 (17.78%)
Frameshift mutation	29 (16.11%)
Nonsense mutation	29 (16.11%)
Mutation classification	
Haploinsufficiency	56 (37.84%)
Dominant negative	92 (62.16%)

### 3.2 There were no significant differences in baseline data between patients with AD and AA

Previous studies have shown that the main causes of death in Marfan syndrome are AD and AA. Therefore, we compared baseline data for patients with AD and AA. A total of 89 patients were included in this analysis. There were 43 ADs with an average age of 33.43 ± 7.87 and 46 AAs with an average age of 33.52 ± 11.07. There was no significant difference between the two groups in the age of onset (*p* = .598). There was no significant difference between the two groups in the diameter of the aorta closely related to AD. The sinus diameter of the AD group was 56.63 ± 10.40, while that of the AA group was 55.38 ± 10.65; the ascending aorta diameter was 53.83 ± 16.87 in the AD group and 46.17 ± 13.09 in the AA group. There was no significant difference in these parameters between the two groups (*p* = .677 and *p* = .085, respectively). Considering that the aortic diameter will increase after the patient experiences an AD, the difference between the two groups will be smaller. In the AD group, the proportion of patients with lens dislocation was relatively small, at approximately 13.95% (*n* = 6), while in the AA group, the proportion was relatively high, at approximately 34.78% (*n* = 16); the difference was significant (*p* = .008). The ratio of myopia in the two groups was relatively high, with 72.09% in the AD group and 65.22% in the AA group; there was no significant difference in this parameter between the two groups (*p* = .485). Mitral valve prolapse is also an important clinical manifestation of patients with MFS and can lead to mitral regurgitation and finally to heart failure, which is also the main cause of death. In this group, there were fewer patients with mitral prolapse: one in the AD group and four in the AA group. There was no significant difference in this parameter between the two groups (*p* = .485).

### 3.3 Frameshift and nonsense mutations have a higher frequency in patients with aortic dissection

**Figure 1:**
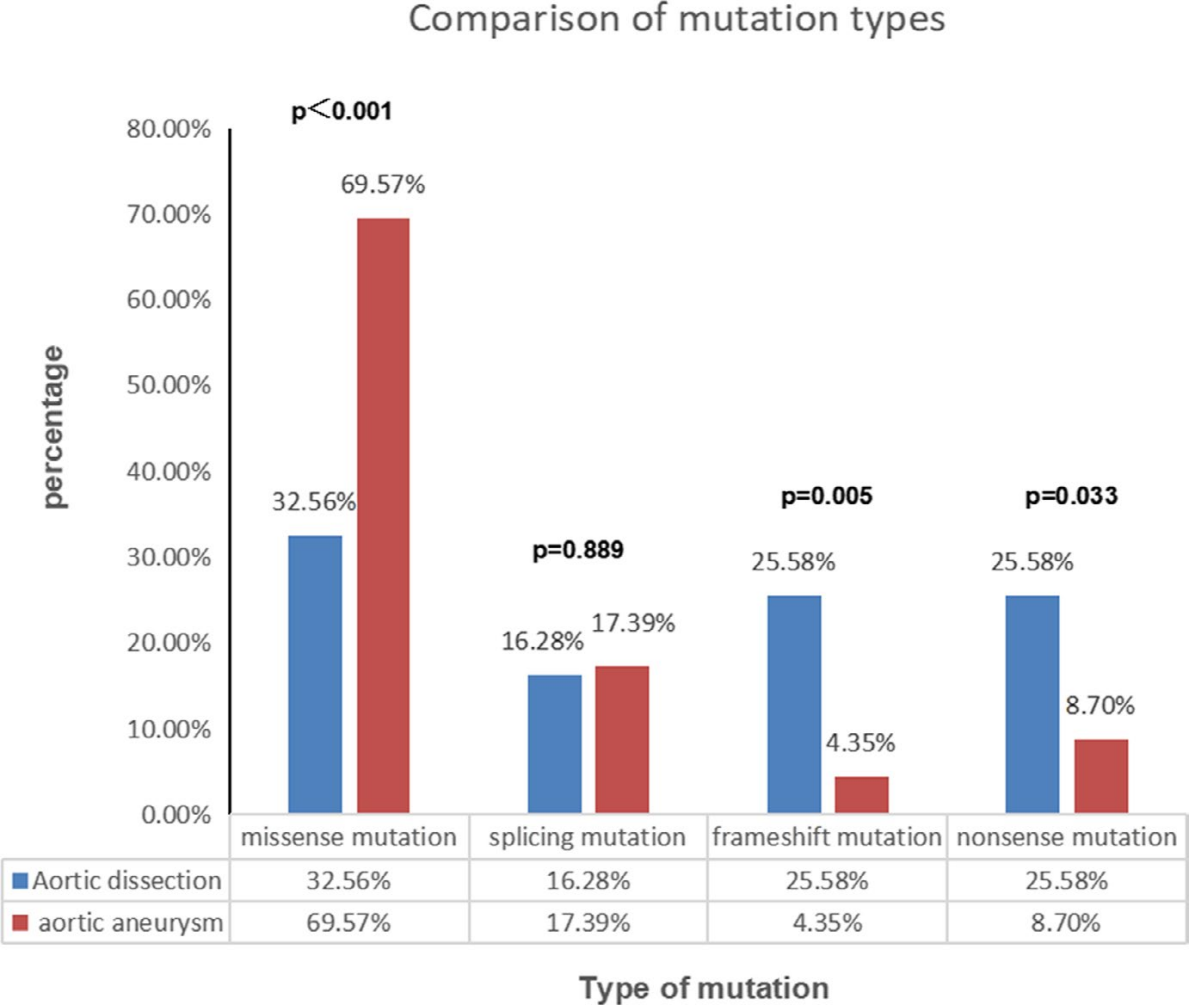
Frameshift and nonsense mutations have a higher frequency in patients with AD. There was a significantly higher frequency of frameshift and nonsense mutations in the AD group than in the AA group (25.58% vs. 4.35%, *p* = .017; 25.58% vs. 8.70%, *p* = .033, respectively), while missense mutations were more frequent in the AA group than in the AD group (69.57% vs. 32.56%, respectively; *p* < .001) and showed a higher rate of lens dislocation (34.78% vs. 13.95%, respectively; *p* = .008)

The most severe manifestations observed in patients with MFS were AD and AA. In addition, some MFS patients had aortic diameters that were not particularly wide when AD occurred. Furthermore, in some patients, the maximum diameter of the aorta reached 90 mm without showing AD. We hypothesized that there might be an intrinsic reason for this more severe clinical phenotype. Therefore, we compared these two groups separately. The results showed that there was no significant difference between the two groups with regard to age at onset or the diameter of the aortic sinus or the ascending aorta, and there was no significant difference in the proportion of males and females. There were significant differences between the two groups in terms of the type of gene mutation. Among the patients with AD, the highest proportion of mutations were missense mutations, accounting for 32.56%, followed by frameshift mutations (25.58%) and nonsense mutations (25.58%), while splicing mutations occurred at a rate of 16.28%. Among the patients with AA, the highest proportion of mutations was missense mutations, accounting for 69.57%, which was significantly higher than that in AD group (69.57% vs. 32.56%, *p* < .001). The proportions of frameshift and nonsense mutations were relatively lower in the AA group than in the AD group (frameshift: 4.35% vs. 25.58%, respectively, *p* = .005; nonsense: 8.70% vs. 25.58%, respectively, *p* = .033). Splicing mutations accounted for 17.39% of mutations in the AA group, which was not significantly different from that in the AD group (17.39% vs. 16.28%, respectively, *p* = .889). The detailed data are provided in Figure 1.

Because phenotypes and genotypes do not show complete cosegregation in MFS patients, the frameshift and nonsense mutations, as well as certain splicing variants, may share the common ending of nonsense‐mediated mRNA decay. Therefore, we divided the variants into haploinsufficiency (HI) and dominant‐negative (DN) groups (Franken et al., 2015). The results showed that most patients with AD were in the HI group (80.77% vs. 31.25%, *p* = .004); there were no significant differences in age of onset, sex, sinus diameter, or ascending aorta diameter between the two groups. The results are shown in Tables S1 and S2.

### 3.4 Comparison of the different genotypes in two different phenotypes in pathological aortic wall specimens

**Figure 2:**
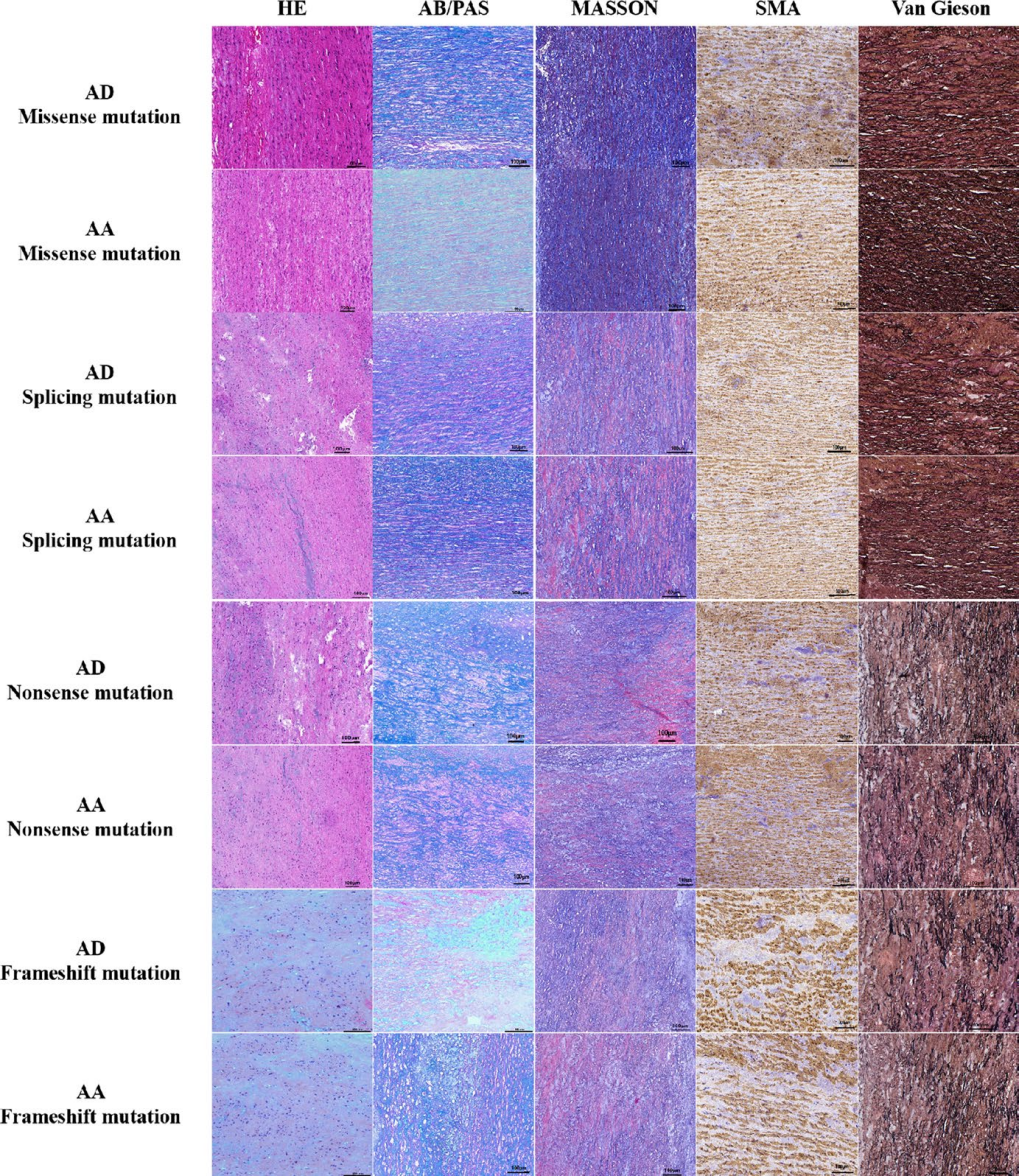
Comparison of the different genotypes in two different phenotypes in pathological aortic wall specimens. The results showed that patients with aortic dissection were more severe than patients with aortic aneurysm. AB/PAS staining showed severe mucosal degeneration in the arterial wall of patients with frameshift and nonsense mutations. MASSON staining showed severe fibrosis. SMA immunohistochemical staining showed that the number of smooth muscle cells was less than that of missense mutations, even partial deletion and disorder, while Van Gieson staining showed that the elastic fibers were obviously broken, flattened, and arranged in a disorderly manner in patients with frameshift or nonsense mutations

To investigate the mechanism by which these two types of FBN1 mutations lead to different clinical phenotypes, we performed HE staining, AB/PAS staining, MASSON staining, SMA staining, and Van Gieson staining on pathological aortic wall specimens obtained from patients who underwent surgery to determine the pathological effects of the different mutation types on the aortic wall between the AD and AA groups (12 patients in AD and 12 patients in AA). The results are shown in Figure 2. The results showed that patients with aortic dissection were more severe than patients with aortic aneurysm; the elastic fiber rupture was more severe, and the smooth muscle cells were fewer in number and were more pronounced and disorganized. Comparing the pathological specimens of aortic wall in four groups of patients with different genotypes, the results showed that the structural damage of aortic wall was more serious in patients with frameshift mutations and nonsense mutations, while the aortic wall lesion was relatively mild in patients with missense mutations. AB/PAS staining showed severe mucosal degeneration in the arterial wall of patients with frameshift and nonsense mutations. MASSON staining showed severe fibrosis in patients with frameshift or nonsense mutations. SMA immunohistochemical staining showed fewer smooth muscle cells in patients with frameshift or nonsense mutations than in patients with missense mutations. Van Gieson staining showed that the elastic fibers were obviously broken, flattened, and arranged in a disorderly manner in patients with frameshift or nonsense mutations (Tables 2 and 3).

**Table 2:** Characteristics between aortic dissection and aortic aneurysm are the same

Variables	Aneurysm (*N* = 46)	Dissection (*N* = 43)	*p*‐value
Basic features			
Age of onset (year)	33.52 ± 11.07	32.43 ± 7.87	0.598
Body mass index (kg/m^2^)	21.21 ± 4.35	22.93 ± 2.60	0.981
Gender (male)	32 (69.57%)	23 (53.49%)	0.119
Family history	25 (54.35%)	18 (41.86%)	0.239
System score	5.00 (4.00–7.00)	5.00 (4.00–7.00)	0.634
Cardiovascular features			
Sinus diameter (mm)	55.38 ± 10.65	56.63 ± 10.40	0.677
Ascending aorta diameter (mm)	46.17 ± 13.09	53.83 ± 16.87	0.085
Descending aorta diameter (mm)	29.00 ± 4.24	28.44 ± 4.69	0.882
Hypertension	1 (2.17%)	4 (9.30%)	0.144
Aortic insufficiency			0.057
None	14 (30.43%)	21 (48.84%)	
Mild	20 (43.48%)	9 (20.93%)	
Moderate	0 (0.00%)	2 (4.65%)	
Severe	12 (26.09%)	11 (25.58%)	
Mitral regurgitation			0.906
None	33 (71.74%)	29 (67.44%)	
Mild	9 (19.57%)	11 (25.58%)	
Moderate	1 (2.17%)	1 (2.33%)	
Severe	3 (6.52%)	2 (4.65%)	
Mitral valve prolapse	4 (8.70%)	1 (2.33%)	0.192
Ocular features			
Lens dislocation	16 (34.78%)	6 (13.95%)	0.008
Myopia	30 (65.22%)	31 (72.09%)	0.485
Skeletal features			
Skeletal deformity	31 (67.39%)	31 (72.09%)	0.630
Wrist sign	34 (73.91%)	31 (72.09%)	0.847
Finger sign	31 (67.39%)	35 (81.40%)	0.132
Wrist and Finger sign	34 (73.91%)	30 (69.77%)	0.664
Thoracic deformity	18 (39.13%)	20 (46.51%)	0.482
Scoliosis	10 (21.74%)	6 (13.95%)	0.339
Lung	4 (8.70%)	5 (11.63%)	0.647
Hernia	4 (8.70%)	1 (2.33%)	0.192

**Table 3:** The frequency of frameshift and nonsense mutations is higher in the aortic dissection group

Variables	Aneurysm (*N* = 46)	Dissection (*N* = 43)	*p*‐value
Type of mutation			
Missense mutation	32 (69.57%)	14 (32.56%)	<0.001
Splicing mutation	8 (17.39%)	7 (16.28%)	0.889
Frameshift mutation	2 (4.35%)	11 (25.58%)	0.005
Nonsense mutation	4 (8.70%)	11 (25.58%)	0.033

## 4 DISCUSSION

In this relatively large sample of studies, by grouping people with different diseases and then analyzing their genotypes, we showed that among patients with MFS, frameshift and nonsense mutations were more common in patients with AD than in patients with AA, while missense mutations were more common in patients with AA than in those with AD; additionally, there were more instances of lens dislocation in patients with AA than in patients with AD. At the same time, we also grouped patients according to their genotypes (Table S3). We found that a high proportion of aortic dissection in MFS patients with FBN1 nonsense and frameshift mutations, predicting that patients with nonsense and frameshift mutations are more prone to aortic dissections. Through the staining of pathological specimens, we showed that there were fewer elastic fibers in the aortic wall and fewer and more disorganized smooth muscle cells in patients with frameshift and nonsense mutations than in patients with missense mutations.

Previous studies have reported that truncating mutations (e.g., frameshift and nonsense mutations) or shear mutations are more likely than missense mutations or mutations involving a replaced cysteine residue to cause AD, but these trends were not significant (Rommel et al., 2005). This result may be due to the small sample size; there were 21 patients with AD and 13 patients with preventive aortic surgery. In our group, the incidence of aortic events was relatively high, with a rate of 49.44%. There were 43 patients with AD and 46 patients with AA. In particular, Schrijver et al. (Schrijver et al., 2002) found that compared to MFS patients with missense mutations (32%; 6/19), in MFS patients with truncating mutations (58%; 15/26; *p* = .08), AD was the main indicator for ascending aorta replacement. This finding is similar to our research. A study by Rommel et al. (Baudhuin, Kotzer, & Lagerstedt, 2015a, 2015b) also found that AD mostly occurred in the protein‐truncation group (5/25; 20%) rather than in the cysteine residue‐replacement group (3/30, 10%). Baudhuin, Linnea M et al. (Baudhuin, Kotzer, & Lagerstedt, 2015a, 2015b) found that the frequencies of truncated or mutated FBN1 gene mutations were higher than the frequency of missense mutations in patients with aortic events. In addition, the authors compared the age of patients and found that the combined rates of truncating and splicing mutations were 100% (*n* = 12) and 95% (*n* = 21) in patients younger than 30 and 40 years old, respectively, and those with truncating or splicing mutations were younger (29 years old) than those with missense mutations (51 years old). We achieved the same results in different populations, but no significant differences were found in terms of age, which may be related to different genetic backgrounds in different populations. Détaint et al. (Baudhuin et al., 2015a, 2015b) found that patients with ascending aortic dilatation, aortic events, and mitral valve prolapse were more likely than other groups to have mutations affecting cysteine residues, but the authors did not analyze correlations between genotypes and clinical phenotypes among patients with AD or prophylactic aortic surgery. Our study found that patients with AA have more lens dislocation than patients with AD (34.78% vs. 13.95%, *p* = .008), consistent with previous findings (Baudhuin et al., 2015a, 2015b).

Patients with MFS appear with age‐dependent symptoms, with approximately 16% of MFS patients under the age of 30 years undergoing AD or prophylactic aortic surgery and approximately 74% of patients under the age of 60 years having an aortic event (AD or AA) (Detaint et al., 2010). These rates are similar to those observed in our study; however, in our patients, aortic events occurred earlier, with approximately 47.50% of the affected patients being less than 30 years old and up to 97.50% being less than 60 years old. This finding may be related to the spectrum of the disease in the Chinese population. In addition, we compared patients of different genders. The results showed that aortic events were more common in male patients than in female patients (61.80% vs. 39.20%, respectively).

The reason that frameshift and nonsense mutations lead to a higher risk of AD in patients with MFS was unclear. We performed a staining analysis of patient‐derived aortic wall samples. Aortic wall elastic fibers were thinner and sparser, and vascular smooth muscle cells were sparser and more disordered in patients with frameshift and nonsense mutations than in those with missense mutations, which may explain why patients with frameshift mutations and nonsense mutations were more prone to AD. One of the effects of MFS was a decrease in the elastic fibers in the aortic wall. This finding was due to mutations in the FBN1 gene that affect fibril proteins. Other effects are mediated by the activation of the TGF‐β signaling pathway (Hillebrand et al., 2014), which leads to increased apoptosis by vascular smooth muscle cells. Compared with missense mutations, frameshift mutations and nonsense mutations had a greater impact on the protein structure of FBN1 and resulted in fewer elastic fibers, weaker elasticity of the extracellular matrix, and eventually AD. Furthermore, frameshift mutations and nonsense mutations may lead to the blockade of FBN1 protein synthesis and, therefore, a decrease in FBN1 protein levels. This decrease leads to the enhanced activation of TGF‐β signaling, which results in increased apoptosis and disordered arrangement of vascular smooth muscle cells, eventually increasing the risk of AD. These findings required further experiments, including in vivo animal experiments, for validation.

An early diagnosis is important for MFS patients. Acute aortic dissection (AAD) due to AA is often the cause of mortality in MFS patients, and AAD patients have a higher reoperative rate even though patients survive the initial surgery (Yang et al., 2016). Importantly, previous studies demonstrated an age‐dependent association with the occurrence of an aortic event (Yang et al., 2016). However, its value in forecasting aortic outcome is currently very limited. The results of our study show that AD occurs more frequently in patients with frameshift mutations and nonsense mutations. This finding suggests that for patients with MFS, if an FBN1 frameshift mutation or nonsense mutation is detected, we should provide more intense surveillance with a stricter imaging protocol and a low threshold for surgery.

In addition, genetic testing appears to be particularly important for an early diagnosis. This test could confirm a diagnosis before symptoms fully develop, thus providing valuable time for prophylactic measures. With proper management, the life expectancy of MFS patients could approximate that of the general population (Vanem et al., 2018). The Revised Ghent criteria also emphasize the importance of genetic sequencing. Compared to the cost of whole‐genome or full‐exome sequencing, panel sequencing is more cost‐effective and capable of identifying hereditary aortic diseases.

In this study, we developed an aortic gene panel that makes DNA sequencing easy, time‐saving, and cost‐effective in this population of patients. Patients with fatal AD were mostly included in the frameshift mutation group in this study. These data provide powerful support for the management and follow‐up of patients with MFS. Of course, this study also has some limitations. The first was that this was a single‐center study and may therefore not be representative of other populations. Later studies should draw on multicenter data. Second, the range of ages of patients with MFS was relatively large and, considering the age‐dependent incidence of MFS, may have resulted in bias. Third, we did not perform an in‐depth study to explore the mechanism by which frameshift mutations and nonsense mutations lead to a higher risk of AD in patients with MFS, and future research is needed to explore this issue.

In general, our study was novel and provided some very important and meaningful findings. In the future, we will perform multicenter studies and use basic experiments to further explore the effects of different types of mutations on the aortic wall in patients with MFS.

## 5 CONCLUSION

The results of this study increase our understanding of the FBN1 gene mutation spectrum in Chinese patients with MFS. We found that frameshift mutations and nonsense mutations were more common in patients with AD and that the aortic wall structure contained fewer elastic fibers and fewer and more disordered smooth muscle cells.

## CONFLICT OF INTEREST

The authors declare they have no competing financial interests.

## AUTHOR CONTRIBUTION

Shijun Xu, Feng Lan, Yihua He, and Hongjia Zhang designed the study. Shijun Xu, Yuwei Fu, and Xin Wang performed the experiments. Hairui Sun and Jianbin Wang performed the data analysis. Shijun Xu wrote the manuscript.

## ETHICAL APPROVAL

This study was approved by the Editorial Policies and Ethical Considerations committee of Beijing Anzhen hospital.

All subjects provided informed consent (for subjects who were younger than 18, consent was signed by their parents), and all information relating to individuals participating in the study was kept strictly confidential.
