# Supplementary material for: Increased frequency of FBN1 frameshift and nonsense mutations in Marfan syndrome patients with aortic dissection
Source: Mol Genet Genomic Med. 2019 Dec 12;8(1):e1041. doi: 10.1002/mgg3.1041 (PMC6978253; doi:10.1002/mgg3.1041)
Supplement: Supplementary file 1 [file MGG3-8-e1041-s001.doc]

## Supplementary table 1：clinical data and FBN1 mutations reported at gene and protein level for each patient.

| **ID** | **Gender** | **Age of onset** | **Age of detection** | **System score** | **Type of mutation** | **Exon** | **Family history** | **Diagnosis** | **Aortic insufficiency** | **Mitral Vve Plapse** | **Lens dislocation** | **Skeletal deformity** | **Thoracic deformity** | **Scoliosis** | **myopia** | **lung** | **hernia** | **Nucleotides** | **Amino acid** |
| --- | --- | --- | --- | --- | --- | --- | --- | --- | --- | --- | --- | --- | --- | --- | --- | --- | --- | --- | --- |
| P101 | male | 44.00 | 44.00 | 7 | missense | 13 | no | aortic dissection | AI.MI | no | no | yes | yes | no | yes | no | no | c.G1511T | p.C504F |
| P103 | male | 32.00 | 32.00 | 1 | splicing | 9 | no | without cardiovascular | no | no | no | no | no | no | yes | no | no | c.2168-1G>T |  |
| P115 | male | 31.00 | 32.00 | 4 | nonsense | 39 | yes | aortic dissection | no | no | no | no | no | no | yes | no | no | c.G4771T | p.G1591X |
| P128 | male | 26.00 | 26.00 | 5 | splicing |  | yes | Aortic aneurysm | AI | no | yes | yes | yes | no | yes | no | no | c.2293+1G>A |  |
| P145 | female | 29.00 | 29.00 | 5 | missense | 58 | yes | without cardiovascular | no | no | no | yes | no | yes | yes | no | no | c.G7094A | p.C2365Y |
| P146 | male | 27.00 | 27.00 | 6 | missense | 58 | yes | Aortic aneurysm | AI | no | yes | yes | yes | yes | yes | no | no | c.G7094A | p.C2365Y |
| P153 | male | 32.00 | 32.00 | 2 | splicing | 20 | yes | aortic dissection | AI | no | yes | no | no | no | yes | no | no | c.2539+1G>C |  |
| P154 | female | 1.25 | 1.25 | 0 | splicing | 20 | yes | without cardiovascular | no | no | no | no | no | no | no | no | no | c.2539+1G>C |  |
| P156 | male | 32.00 | 32.00 | 6 | splicing | 15 | yes | aortic dissection | AI | no | no | yes | yes | no | yes | no | no | c.1715-1G>C |  |
| P157 | female | 1.70 | 1.70 | 1 | splicing | 15 | yes | Sinus width | no | no | no | no | no | no | no | no | no | c.1715-1G>C |  |
| P158 | female | 50.00 | 50.00 | 1 | missense | 41 | yes | Cardiovascular performanc | no | no | no | no | no | no | yes | no | no | c.T5059C | p.C1687R |
| P159 | male | 26.00 | 26.00 | 2 | missense | 41 | yes | Aortic aneurysm | no | no | yes | yes | yes | no | yes | no | no | c.T5059C | p.C1687R |
| P178 | male | 3.00 | 18.00 | 8 | missense | 34 | no | Cardiovascular performanc | MI.TI | no | yes | yes | yes | no | yes | no | no | c.T4138A | p.C1380S |
| P185 | female | 32.00 | 35.00 | 5 | frameshift | 28 | no | aortic dissection | no | no | no | yes | no | no | yes | no | no | c.3451_3452del | p.S1151Rfs*7 |
| P187 | male | 5.50 | 5.50 | 3 | frameshift | 28 | yes | without cardiovascular | no | no | no | yes | no | yes | no | no | no | c.3451_3452del | p.S1151Rfs*7 |
| P193 | male | 23.00 | 27.00 | 5 | missense | 52 | yes | Cardiovascular performanc | MI.TI | no | no | yes | yes | no | yes | no | no | c.T6331G | p.C2111G |
| P194 | female | 5.00 | 5.00 | 4 | missense | 52 | yes | Cardiovascular performanc | no | no | no | no | no | no | no | no | no | c.T6331G | p.C2111G |
| P197 | male | 0.20 | 0.20 | 7 | splicing | 26 | no | without cardiovascular | no | no | no | yes | yes | no | no | no | no | c.2677+1G>A |  |
| P201 | female | 27.00 | 27.00 | 7 | nonsense | 58 | no | Sinus width | no | no | no | yes | no | yes | yes | yes | no | c.C7180T | p.R2394X |
| P205 | male | 24.00 | 24.00 | 4 | missense | 64 | no | Sinus width | no | no | no | yes | no | yes | yes | no | no | c.G7963A | p.A2655T |
| P208 | male | 19.00 | 27.00 | 7 | missense | 22 | no | Aortic aneurysm | no | no | yes | yes | yes | yes | yes | no | no | c.G2638A | p.G880S |
| P231 | male | 34.00 | 34.00 | 2 | missense | 53 | no | Aortic aneurysm | no | no | no | no | no | no | no | no | no | c.A6431G | p.N2144S |
| P235 | male | 24.00 | 24.00 | 4 | frameshift | 28 | yes | Sinus width | no | no | no | no | no | no | no | no | no | c.3380_3381insA | p.V1128Cfs*4 |
| P238 | male | 24.00 | 24.00 | 5 | missense | 20 | no | Aortic aneurysm | no | no | yes | yes | yes | no | yes | no | no | c.T2374C | p.C792R |
| P239 | female | 30.00 | 30.00 | 5 | missense | 14 | yes | Aortic aneurysm | AI | no | no | yes | no | yes | no | no | no | c.G1622A | p.C541Y |
| P244 | female | 24.00 | 26.00 | 2 | missense | 19 | yes | aortic dissection | AI.MI.TI | yes | no | no | no | no | yes | no | no | c.G2243A | p.C748Y |
| p245 | male | 20.00 | 20.00 | 2 | missense | 19 | yes | Aortic aneurysm | no | no | no | yes | yes | no | yes | no | no | c.G2243A | p.C748Y |
| P252 | male | 35.00 | 35.00 | 2 | missense | 4 | no | without cardiovascular | no | no | yes | yes | no | no | yes | no | no | c.T278G | p.F93C |
| P253 | male | 3.00 | 3.00 | 3 | missense | 4 | yes | without cardiovascular | no | no | yes | yes | yes | no | yes | no | no | c.T278G | p.F93C |
| P259 | female | 48.00 | 48.00 | 0 | missense | 16 | yes | Sinus width | no | no | no | no | no | no | no | no | no | c.A1838G | p.D613G |
| P260 | male | 25.00 | 25.00 | 4 | missense | 16 | yes | Aortic aneurysm | AI | no | no | no | no | no | yes | no | no | c.A1838G | p.D613G |
| P261 | male | 30.00 | 30.00 | 7 | missense | 41 | yes | aortic dissection | AI | no | no | no | yes | yes | yes | no | no | c.T5036G | p.M1679R |
| P262 | female | 6.00 | 6.00 | 4 | missense | 41 | yes | without cardiovascular | no | no | no | yes | no | no | yes | no | no | c.T5036G | p.M1679R |
| P263 | male | 7.00 | 7.00 | 0 | missense | 41 | yes | without cardiovascular | no | no | no | no | no | no | no | no | no | c.T5036G | p.M1679R |
| P264 | female | 25.00 | 25.00 | 7 | splicing | 19 | yes | Aortic aneurysm | AI.MI | yes | no | yes | no | no | yes | yes | no | c.2293+1G>A |  |
| P282 | male | 36.00 | 36.00 | 6 | splicing | 30 | no | Aortic aneurysm | AI | no | no | yes | no | no | no | yes | yes | c.3590-1G>A |  |
| P285 | male | 11.00 | 11.00 | 5 | splicing | 30 | yes | without cardiovascular | no | no | no | yes | no | no | yes | no | no | c.3590-1G>A |  |
| P290 | female | 5.00 | 5.00 | 2 | missense | 15 | no | Cardiovascular performanc | no | no | yes | yes | no | yes | yes | no | no | c.G1837A | p.D613N |
| P300 | male | 31.00 | 36.00 | 4 | missense |  | no | Aortic aneurysm | AI | no | yes | yes | no | no | yes | no | no | c.G1837A | p.D613N |
| P726 | male | 32.00 | 32.00 | 5 | missense | 22 | yes | Aortic aneurysm | no | no | yes | no | no | no | yes | no | no | c.C2645T | p.A882V |
| P727 | male | 38.00 | 57.00 | 4 | missense | 22 | no | Aortic dissection | AI | no | no | yes | no | no | yes | no | no | c.C2645T | p.A882V |
| P728 | male | 32.00 | 32.00 | 3 | missense | 22 | yes | Aortic aneurysm | AI | no | no | no | no | no | yes | no | no | c.C2645T | p.A882V |
| A001226-1_P02_O | male | 26.00 | 26.00 | 3 | missense | 22 | yes | Aortic aneurysm | AI | no | no | yes | yes | no | yes | no | no | c.C2645T | p.A882V |
| A001094-1_P02_O | female | 9.00 | 9.00 | 5 | missense | 4 | no | Sinus width | no | no | yes | yes | no | no | no | no | no | c.G332A | p.C111Y |
| A001329-1_P02_O | female | 28.00 | 35.00 | 5 | nonsense | 29 | yes | Aortic dissection | no | no | no | yes | yes | no | no | no | no | c.C3546A | p.C1182X |
| A001329-3_P02_M | female | 53.00 | 53.00 | 3 | nonsense | 29 | no | Aortic dissection | no | no | no | no | no | no | no | no | no | c.C3546A | p.C1182X |
| P320 | female | 4.00 | 5.00 | 8 | missense | 32 | no | Sinus width | no | no | yes | no | yes | no | no | no | no | c.G3851A | p.C1284Y |
| P683 | female | 26.00 | 31.00 | 6 | missense | 32 | yes | Aortic aneurysm | AI | no | yes | yes | no | no | yes | no | no | c.G3872A | p.C1291Y |
| P685 | female | 8.00 | 8.00 | 4 | missense | 32 | yes | Sinus width | no | no | no | yes | no | no | yes | no | no | c.G3872A | p.C1291Y |
| P574 | male | 24.00 | 24.00 | 7 | missense | 33 | no | Aortic aneurysm | no | no | no | yes | yes | no | no | no | no | c.T4017G | p.C1339W |
| P687 | female | 37.00 | 37.00 | 6 | missense | 35 | no | Aortic aneurysm | AI | no | yes | yes | no | no | yes | no | no | c.T4222C | p.C1408R |
| A000843-1_P02_O | female | 19.00 | 19.00 | 3 | missense | 35 | no | Sinus width | no | no | yes | no | no | no | no | no | no | c.T4222C | p.C1408R |
| A000843-2_P02_SON | male |  |  | 5 | missense | 35 | yes | Sinus width | no | no | yes | no | no | no | no | no | no | c.T4222C | p.C1408R |
| A001057-1_P02_O | male | 4.50 | 4.50 | 4 | missense | 35 | yes | Sinus width | no | no | yes | no | no | no | no | no | yes | c.C4260G | p.C1420W |
| A000560-1_P02_O | female | 21.00 | 38.00 | 7 | missense | 35 | no | Aortic aneurysm | no | no | no | no | no | no | no | no | no | c.T4285C | p.C1429R |
| P729 | female | 34.00 | 36.00 | 5 | missense | 37 | no | Aortic dissection | no | no | yes | no | no | no | yes | no | no | c.G4472A | p.C1491Y |
| P731 | male | 9.00 | 9.00 | 5 | missense | 37 | yes | Sinus width | no | no | yes | yes | no | no | yes | no | no | c.G4472A | p.C1491Y |
| A001387-1_P02_O | male | 54.00 | 56.00 | 5 | missense | 6 | no | Aortic aneurysm | AI | no | no | yes | no | no | yes | no | no | c.T496A | p.C166S |
| A001486-1_P02_O | male | 36.00 | 41.00 | 5 | missense | 42 | no | Aortic aneurysm | AI | no | no | yes | no | no | no | no | no | c.G5156A | p.C1719Y |
| A001486-4_P02_DAU | female | 19.00 | 19.00 | 4 | missense | 42 | yes | without cardiovascular | no | no | no | yes | no | no | no | no | no | c.G5156A | p.C1719Y |
| A001217-1_P02_O | female | 3.00 | 5.00 | 3 | nonsense | 47 | no | without cardiovascular | no | no | yes | yes | yes | no | no | no | no | c.C5715A | p.C1905X |
| A001199-1_P02_O | female | 19.00 | 26.00 | 9 | missense | 50 | yes | Mitral valve prolapse | no | yes | no | yes | yes | yes | yes | yes | no | c.T6112C | p.C2038R |
| A001362-1_P02_O | female | 8.00 | 8.00 | 2 | nonsense | 53 | no | Mitral valve prolapse | no | yes | no | no | no | no | no | no | no | c.C6426A | p.C2142X |
| P325 | male | 38.00 | 38.00 | 4 | missense | 59 | yes | Sinus width | no | no | no | no | no | no | yes | no | yes | c.G7238A | p.C2413Y |
| P_325 | male | 38.00 | 38.00 | 5 | missense | 59 | no | Sinus width | AI | no | yes | yes | no | no | yes | no | yes | c.G7238A | p.C2413Y |
| P575 | male | 52.00 | 52.00 | 7 | missense | 60 | no | aortic dissection | no | no | no | no | no | no | no | no | no | c.T7363G | p.C2455G |
| P763 | female | 22.00 | 22.00 | 5 | nonsense | 60 | no | without cardiovascular | no | no | no | yes | yes | no | no | no | no | c.C7410A | p.C2470X |
| A001587-1_P02_O | male | 29.00 | 29.00 | 6 | missense | 62 | yes | Sinus width | no | yes | no | yes | yes | no | no | no | no | c.G7649A | p.C2550Y |
| P666 | female | 25.00 | 25.00 | 5 | missense | 63 | no | Aortic dissection | no | no | no | yes | yes | no | no | no | no | c.G7712A | p.C2571Y |
| A001651-1_P02_O | female | 34.00 | 34.00 | 4 | missense | 13 | no | Aortic dissection | no | no | no | yes | no | no | no | no | no | c.G1481A | p.C494Y |
| P321 | female | 29.00 | 34.00 | 4 | missense | 13 | yes | aortic dissection | no | no | no | yes | no | no | yes | yes | no | c.G1511A | p.C504Y |
| P323 | male | 26.00 | 26.00 | 4 | missense | 13 | yes | Aortic aneurysm | no | no | no | no | no | no | yes | no | no | c.G1511A | p.C504Y |
| P472 | male | 14.00 | 14.00 | 6 | frameshift | 64 | yes | without cardiovascular | no | no | no | yes | no | yes | yes | no | yes | c.7881delG | p.S2628Afs*54 |
| P650 | male | 12.00 | 12.00 | 4 | missense | 14 | no | without cardiovascular | no | no | yes | no | no | no | yes | no | no | c.T1708C | p.C570R |
| A001662-4_P02_OM | male | 26.00 | 26.00 | 6 | missense | 15 | no | Aortic aneurysm | AI | no | yes | yes | yes | no | yes | no | yes | c.G1832A | p.C611Y |
| P580 | male | 28.00 | 28.00 | 4 | nonsense | 19 | no | aortic dissection | AI | no | no | yes | yes | no | no | no | no | c.C2202A | p.C734X |
| A000808-1_P02_O | female | 5.00 | 6.00 | 6 | missense | 24 | no | Sinus width | no | yes | yes | yes | no | no | no | no | no | c.T2809C | p.C937R |
| A001298-1_P02_O | female | 29.00 | 33.00 | 2 | missense | 48 | no | Aortic dissection | no | no | yes | no | no | no | yes | no | no | c.T85824C | p.Cys1942Arg |
| A001366-1_P02_O | male | 3.00 | 3.00 | 6 | splicing | 27 | no | Sinus width | no | yes | yes | yes | no | no | yes | no | no | c.G3337T |  |
| A001210-1_P02_O | female | 41.00 | 41.00 | 4 | missense | 34 | yes | Aortic aneurysm | AI | no | yes | no | no | no | yes | no | no | c.A4088G | p.D1363G |
| A001210-2_P02_DAU | female | 20.00 | 20.00 | 4 | missense | 34 | yes | without cardiovascular | no | no | no | yes | no | no | yes | no | no | c.A4088G | p.D1363G |
| A001210-3_P02_YS | female | 32.00 | 32.00 |  | missense | 34 | yes | Sinus width | no | no | no | no | no | no | no | no | no | c.A4088G | p.D1363G |
| A001210-4_P02_ZN | female | 9.00 | 9.00 |  | missense | 34 | yes | Sinus width | no | no | no | no | no | no | no | no | no | c.A4088G | p.D1363G |
| A001453-1_P02_O | female | 31.00 | 31.00 | 6 | nonsense | 37 | no | Aortic dissection | no | no | yes | yes | no | no | yes | no | no | c.G4552T | p.E1518X |
| P405 | male | 35.00 | 35.00 | 8 | nonsense | 38 | yes | aortic dissection | AI.MI | no | no | yes | yes | yes | yes | no | no | c.G4723T | p.E1575X |
| A001318-1_P02_O | female | 32.00 | 32.00 | 5 | nonsense | 39 | yes | without cardiovascular | no | no | no | yes | no | no | no | no | no | c.G4750T | p.E1584X |
| A001318-4_P02_SON | male | 3.00 | 3.00 | 1 | nonsense | 39 | yes | Sinus width | no | no | no | no | no | no | no | no | no | c.G4750T | p.E1584X |
| P671 | female | 31.00 | 30.00 | 4 | missense | 60 | yes | Sinus width | no | no | no | yes | no | no | no | no | no | c.A7340G | p.E2447G |
| P672 | male | 30.00 | 31.00 | 1 | missense | 60 | no | without cardiovascular | no | no | no | no | no | no | yes | no | no | c.A7340G | p.E2447G |
| P302 | male | 47.00 | 57.00 | 5 | frameshift | 62 | yes | aortic dissection | no | no | no | no | no | no | no | yes | no | c.7581delA | p.E2527Dfs*155 |
| P303 | male | 28.00 | 31.00 | 4 | frameshift | 62 | yes | Cardiovascular performanc | no | no | no | yes | no | no | yes | no | no | c.7581delA | p.E2527Dfs*155 |
| P304 | female | 9.00 | 9.00 | 0 | frameshift | 62 | yes | without cardiovascular | no | no | no | no | no | no | no | no | no | c.7581delA | p.E2527Dfs*155 |
| P305 | male | 37.00 | 37.00 | 5 | frameshift | 62 | yes | without cardiovascular | no | no | no | yes | yes | no | yes | no | no | c.7581delA | p.E2527Dfs*155 |
| P306 | female | 32.00 | 32.00 | 5 | frameshift | 62 | yes | without cardiovascular | no | no | no | yes | no | no | no | no | no | c.7581delA | p.E2527Dfs*155 |
| P489 | male | 32.00 | 32.00 | 6 | frameshift | 66 | no | aortic dissection | AI.MI.TI | no | no | yes | yes | yes | yes | yes | no | c.8573delT | p.L2858Rfs*5 |
| P471 | female | 29.00 | 32.00 | 5 | splicing | 64 | no | aortic dissection | no | no | no | yes | no | no | yes | no | no | c.1715-1G>C |  |
| P470 | female | 29.00 | 32.00 | 7 | frameshift | 64 | yes | Aortic dissection | no | no | no | yes | yes | no | yes | no | no | c.7881delG | p.S2628Afs*54 |
| P472 | male | 14.00 | 14.00 | 7 | frameshift | 64 | yes | without cardiovascular | no | no | no | yes | yes | yes | yes | no | no | c.7881delG | p.S2628Afs*54 |
| P646 | male | 32.00 | 32.00 | 5 | frameshift | 7 | no | aortic dissection | no | no | no | yes | yes | yes | yes | no | no | c.1506delT | p.E503Sfs*76 |
| P646 | male | 36.00 | 25.00 | 7 | frameshift | 13 | yes | Aortic dissection | AI | no | no | yes | yes | no | yes | no | no | c.1506delT | p.E503Sfs*76 |
| A000533-1_P02_O | male | 27.00 | 27.00 | 7 | missense | 22 | yes | Aortic aneurysm | AI | no | no | yes | yes | no | no | no | no | c.G2639A | p.G880D |
| A000533-3_P02_M | female | 52.00 | 52.00 | 7 | missense | 22 | yes | Sinus width | no | yes | no | yes | yes | no | no | no | no | c.G2639A | p.G880D |
| P579 | male | 24.00 | 24.00 | 4 | missense | 22 | yes | aortic dissection | AI | no | no | yes | yes | no | no | no | no | c.G2638A | p.G880S |
| A000458-1_P02_O | male | 28.00 | 28.00 | 7 | missense | 23 | no | Sinus width | no | no | no | yes | no | yes | yes | no | no | c.G2696T | p.G899V |
| A000458-3_P02_DAU | female | 1.80 | 1.80 | 3 | missense | 23 | yes | Sinus width | no | yes | no | no | no | no | no | no | no | c.G2696T | p.G899V |
| P317 | male | 22.00 | 28.00 | 6 | frameshift | 36 | no | aortic dissection | no | no | no | no | yes | no | yes | no | no | c.4420_4423del | p.I1474Afs*14 |
| P519 | female | 26.00 | 26.00 | 3 | missense | 56 | no | aortic dissection | no | no | no | yes | no | no | yes | no | no | c.T6806C | p.I2269T |
| P703 | male | 33.00 | 33.00 | 4 | missense | 24 | no | Aortic aneurysm | AI | no | no | no | no | no | no | yes | yes | c.T2846A | p.I949N |
| P543 | female | 27.00 | 27.00 | 4 | missense | 35 | no | Aortic aneurysm | no | no | yes | no | no | no | yes | no | no | c.T4262C | p.L1421P |
| P783 | male | 25.00 | 33.00 | 7 | frameshift | 64 | no | Aortic dissection | AI | no | no | yes | yes | no | yes | no | no | c.8011_8012del | p.L2671Vfs*33 |
| P786 | male | 35.00 | 36.00 | 3 | frameshift | 66 | no | Aortic aneurysm | no | no | no | no | no | no | yes | no | no | c.8525_8529del | p.L2842Pfs*7 |
| P475 | male | 5.00 | 6.00 | 2 | missense | 14 | yes | Sinus width | no | no | no | yes | no | no | yes | no | no | c.G3038T | p.G1013V |
| A001342-1-P02-0 | female | 31.00 | 31.00 | 7 | frameshift | 31 | no | Sinus width | no | no | no | yes | yes | no | yes | no | no | c.3823_3830del | p.M1275Lfs*6 |
| A001342-2_P02_SON | male | 4.00 | 4.00 | 5 | frameshift | 31 | yes | Sinus width | no | no | no | yes | yes | no | no | no | no | c.3823_3830del | p.M1275Lfs*6 |
| P546 | male | 25.00 | 36.00 | 7 | frameshift | 64 | yes | aortic dissection | no | no | no | yes | yes | no | yes | no | no | c.667_668insGA | p.M223Rfs*108 |
| P547 | male | 6.00 | 6.00 | 7 | frameshift | 7 | yes | Sinus width | no | no | no | yes | yes | no | no | no | no | c.667_668insGA | p.M223Rfs*108 |
| P495 | male | 0.80 | 0.80 | 10 | missense | 25 | no | Cardiovascular performanc | MI | no | no | yes | no | no | no | yes | yes | c.G3038T | p.G1013V |
| P503 | female | 41.00 | 41.00 | 7 | frameshift | 58 | yes | Aortic aneurysm | no | no | yes | yes | yes | no | no | no | no | c.7039_7040del | p.M2347Vfs*19 |
| P767 | male | 62.00 | 62.00 | 5 | missense | 37 | yes | Aortic aneurysm | AI | no | yes | yes | no | no | yes | no | no | c.A4511G | p.N1504S |
| P767 | male | 61.00 | 62.00 | 4 | missense | 37 | yes | Aortic aneurysm | AI | no | no | yes | no | no | yes | no | no | c.A4511G | p.N1504S |
| A001214-1_P02_O | male | 14.00 | 14.00 | 6 | frameshift | 55 | no | without cardiovascular | no | no | no | yes | yes | no | no | no | yes | c.6623delA | p.N2208Mfs*15 |
| P667 | male | 18.00 | 18.00 | 7 | missense | 64 | yes | Sinus width | no | no | no | yes | yes | yes | yes | no | no | c.C7827G | p.N2609K |
| P769 | female | 36.00 | 36.00 | 7 | frameshift | 3 | yes | Sinus width | no | no | no | yes | yes | no | yes | yes | no | c.234delT | p.Q79Sfs*29 |
| A000437-2 | female | 9.00 | 9.00 | 8 | frameshift | 3 | yes | Sinus width | no | yes | yes | yes | yes | yes | yes | no | no | c.234delT | p.Q79Sfs*29 |
| P390 | male | 24.00 | 24.00 | 9 | missense | 38 | no | aortic dissection | AI | no | no | yes | yes | yes | no | no | no | c.C4588T | p.R1530C |
| P397 | female | 44.00 | 44.00 | 5 | missense | 38 | yes | aortic dissection | AI | no | yes | yes | yes | no | yes | no | no | c.C4588T | p.R1530C |
| P407 | female | 18.00 | 18.00 | 5 | missense | 38 | yes | Sinus width | no | no | no | no | no | no | no | no | no | c.C4588T | p.R1530C |
| P527 | female | 44.00 | 44.00 | 1 | nonsense | 39 | yes | Aortic aneurysm | AI.MI | yes | no | no | no | no | yes | no | no | c.C4786T | p.R1596X |
| P529 | female | 17.00 | 17.00 | 4 | nonsense | 39 | yes | Sinus width | no | no | no | no | no | no | yes | no | no | c.C4786T | p.R1596X |
| P504 | female | 16.00 | 16.00 | 6 | frameshift | 58 | yes | Sinus width | no | no | no | yes | yes | yes | yes | no | no | c.7039_7040del | p.M2347Vfs*19 |
| P507 | female | 40.00 | 40.00 | 2 | missense | 47 | yes | without cardiovascular | no | no | yes | yes | no | no | yes | no | no | c.G5744A | p.R1915H |
| P509 | female | 14.00 | 14.00 | 3 | missense | 47 | yes | without cardiovascular | no | no | no | no | no | yes | yes | no | no | c.G5744A | p.R1915H |
| P510 | male | 0.30 | 0.30 | 0 | missense | 47 | yes | Cardiovascular performanc | no | no | no | no | no | no | no | no | no | c.G5744A | p.R1915H |
| P511 | female | 38.00 | 38.00 | 1 | missense | 47 | yes | without cardiovascular | no | no | no | no | no | no | yes | no | no | c.G5744A | p.R1915H |
| P512 | male | 64.00 | 64.00 | 0 | missense | 47 | yes | Aortic aneurysm | no | no | no | no | no | no | no | no | no | c.G5744A | p.R1915H |
| A001384-1_P02_O | female | 48.00 | 48.00 | 3 | frameshift | 48 | no | Aortic dissection | AI | no | no | yes | no | no | yes | no | no | c.5906_5907insAG | p.T1970Gfs*11 |
| A001384-2_P02_DAU | female | 21.00 | 21.00 | 4 | frameshift | 48 | no | without cardiovascular | no | no | no | yes | no | no | yes | no | no | c.5906_5907insAG | p.T1970Gfs*11 |
| A000723-1_P02_O | female | 29.00 | 29.00 | 7 | missense | 53 | yes | Sinus width | no | no | no | yes | yes | yes | no | no | no | c.G6449C | p.R2150P |
| A000723-2_P02_M | female | 52.00 | 54.00 | 5 | missense | 53 | no | Aortic aneurysm | AI | no | no | yes | no | yes | no | no | no | c.G6449C | p.R2150P |
| A001457-1_P02_O | male | 30.00 | 30.00 | 8 | nonsense | 7 | no | Aortic aneurysm | AI | no | no | yes | yes | yes | yes | no | no | c.C643T | p.R215X |
| A001457-3_P02_SON | male | 8.00 | 8.00 | 4 | nonsense | 7 | yes | Mitral valve prolapse | no | yes | no | yes | yes | no | no | no | no | c.C643T | p.R215X |
| A001322-1_P02_O | female | 33.00 | 33.00 | 4 | nonsense | 58 | yes | Aortic dissection | AI | no | no | yes | no | no | no | no | no | c.C7180T | p.R2394X |
| A001322-3_P02_DAU | female | 11.00 | 11.00 | 3 | nonsense | 58 | yes | Sinus width | no | yes | no | yes | no | no | no | no | no | c.C7180T | p.R2394X |
| A001654-1_P02_O | male | 35.00 | 35.00 | 6 | missense | 59 | yes | Aortic aneurysm | AI | no | no | yes | no | yes | no | no | no | c.G5156A | p.C1719Y |
| P569 | male | 39.00 | 39.00 | 7 | missense | 63 | no | Aortic aneurysm | AI.MI | yes | no | yes | yes | yes | no | no | no | c.C7726T | p.R2576C |
| P515 | female | 32.00 | 33.00 | 3 | nonsense | 65 | yes | aortic dissection | AI | no | no | yes | no | no | yes | no | no | c.C8080T | p.R2694X |
| P518 | male | 5.00 | 5.00 | 1 | missense | 65 | yes | Sinus width | no | no | no | yes | yes | no | no | no | no | c.A4088G | p.D1363G |
| P709 | male | 7.00 | 7.00 | 4 | nonsense | 65 | yes | without cardiovascular | no | no | no | yes | yes | yes | no | no | no | c.C8080T | p.R2694X |
| P710 | male | 5.00 | 5.00 | 1 | nonsense | 65 | yes | without cardiovascular | no | no | no | yes | yes | yes | no | no | no | c.C8080T | p.R2694X |
| P391 | male | 34.00 | 34.00 | 4 | nonsense | 11 | no | Aortic aneurysm | AI | no | no | no | no | yes | no | no | no | c.C1285T | p.R429X |
| P421 | female | 10.00 | 10.00 | 7 | nonsense | 11 | no | Sinus width | no | no | no | yes | yes | no | yes | no | no | c.C1285T | p.R429X |
| P570 | male | 27.00 | 27.00 | 7 | nonsense | 11 | no | Aortic aneurysm | AI.MI | no | no | yes | yes | yes | yes | no | no | c.C1285T | p.R429X |
| P656 | male | 27.00 | 27.00 | 3 | nonsense | 13 | yes | Sinus width | AI | no | no | no | no | no | yes | yes | yes | c.C1546T | p.R516X |
| P657 | female | 3.00 | 3.00 | 2 | nonsense | 13 | yes | without cardiovascular | no | no | no | no | no | no | no | no | no | c.C1546T | p.R516X |
| P658 | male | 44.00 | 44.00 | 7 | nonsense | 13 | no | aortic dissection | no | no | no | yes | no | no | yes | yes | no | c.C1546T | p.R516X |
| P602 | male | 46.00 | 46.00 | 2 | missense | 62 | yes | Aortic aneurysm | no | no | no | yes | no | no | yes | no | no | c.C7610G | p.S2537C |
| P603 | male | 35.00 | 35.00 | 3 | missense | 62 | yes | Aortic aneurysm | no | no | no | no | no | no | no | no | yes | c.C7610G | p.S2537C |
| P410 | male | 24.00 | 29.00 | 7 | frameshift | 31 | no | aortic dissection | no | no | no | yes | yes | no | yes | yes | no | c.3836dupT | p.D1280Rfs*4 |
| P764 | female | 27.00 | 32.00 | 3 | nonsense | 42 | no | Aortic dissection | AI | no | no | no | no | no | no | no | no | c.T5100G | p.Y1700X |
| A000874-1_P02_O | male |  |  | 8 | nonsense | 60 | no | Aortic dissection | no | no | no | yes | yes | yes | yes | no | yes | c.7397dupA | p.Y2466_Q2467delinsX |
| P312 | female | 29.00 | 29.00 | 7 | splicing | 19 | yes | without cardiovascular | no | no | yes | yes | no | yes | yes | no | no | c.2293+1G>A |  |
| P514 | male | 6.00 | 6.00 | 4 | splicing | 37 | yes | Sinus width | no | no | no | yes | yes | no | no | no | no | c.4460-2A>G |  |
| P514 | male | 6.00 | 6.00 | 2 | splicing | 37 | yes | Sinus width | no | no | no | yes | yes | no | no | no | no | c.4460-2A>G |  |
| P577 | female | 25.00 | 25.00 | 7 | splicing | 19 | yes | Aortic aneurysm | AI | yes | no | yes | yes | no | yes | yes | no | c.2293+1G>A |  |
| P604 | female | 44.00 | 44.00 | 7 | splicing |  | no | Aortic aneurysm | AI | no | yes | yes | yes | no | yes | no | no | c.5788+5G>T |  |
| P605 | female | 21.00 | 21.00 | 7 | splicing |  | yes | Aortic aneurysm | AI | no | yes | yes | yes | yes | yes | no | no | c.5788+5G>T |  |
| P744 | male | 26.00 | 28.00 | 4 | splicing | 7 | no | Aortic dissection | AI | no | no | yes | yes | no | yes | no | no | c.736+1G>A |  |
| P745 | male | 2.00 | 2.00 | 1 | splicing | 7 | yes | Sinus width | no | no | no | yes | no | no | no | no | no | c.736+1G>A |  |
| P674 | female | 1.00 | 1.00 | 3 | splicing |  | yes | Sinus width | no | no | no | yes | no | no | no | no | no | c.7699+5G>T |  |
| P675 | male | 26.00 | 28.00 | 2 | splicing |  | yes | Aortic dissection | AI | no | no | yes | no | no | no | no | no | c.7699+5G>T |  |
| A000530-1_P02_O | male | 27.00 | 28.00 | 1 | splicing | 13 | no | Aortic aneurysm | AI | no | no | no | no | no | no | no | no | c.1588+1G>A |  |
| A000530-2_P02_SON | male | 5.00 | 5.00 | 1 | splicing | 13 | yes | Sinus width | no | no | no | no | no | no | no | no | no | c.1588+1G>A |  |
| A000564-1_P02_O | female | 4.00 | 4.00 | 3 | splicing | 18 | no | Sinus width | no | yes | yes | no | no | no | yes | no | no | c.2167+1G>A |  |
| A000663_P02_T | female | 26.00 | 26.00 | 7 | splicing | 44 | no | Aortic dissection | no | no | yes | yes | no | no | yes | no | no | c.5297-1G>T |  |
| A000726_P02_T | female | 18.00 | 18.00 | 9 | splicing | 15 | yes | Sinus width | no | yes | yes | yes | yes | no | yes | no | no | c.1837+2T>C |  |
| A000747-1_P02_O | male | 36.00 | 47.00 | 2 | splicing | 60 | yes | Aortic dissection | AI | no | no | no | no | no | yes | no | no | c.7331-1G>T |  |
| A000807-1_P02_O | female | 35.00 | 40.00 | 6 | splicing | 30 | no | without cardiovascular | no | no | yes | yes | yes | no | yes | no | no | c.3712+1G>T |  |
| A001220-1_P02_O | female | 11.00 | 11.00 | 8 | splicing | 53 | no | Sinus width | no | yes | yes | yes | yes | no | yes | no | no | c.6496+1G>T |  |
| A001491-1_P02_O | female | 29.00 | 29.00 | 7 | splicing | 47 | yes | Aortic aneurysm | no | no | no | yes | yes | no | yes | no | no | c.5788+1G>A |  |
| A001491-3_P02_DAU | female | 7.00 | 7.00 | 7 | splicing | 47 | yes | without cardiovascular | no | no | yes | yes | yes | no | yes | no | no | c.5788+1G>A |  |

## Supplementary table 2: Characteristics between Haploinsufficiency and Dominant negative are the same.

| **Variables** | **Haploinsufficiency(N=56)** | **Dominant negative(N=92)** | ***P*-value** |
| --- | --- | --- | --- |
| **Basic features** |  |  |  |
| Age of onset (year) | 27.00 (10.50-32.00) | 27.00 (19.00-35.00) | 0.233 |
| Body mass index (kg/m^2^) | 20.51±3.91 | 19.72±4.67 | 0.310 |
| Gender |  |  | 0.367 |
| femal | 28 (50.00%) | 39 (42.39%) |  |
| male | 28 (50.00%) | 53 (57.61%) |  |
| Family history | 33 (58.93%) | 54 (58.70%) | 0.978 |
| System score | 5.00 (3.75-7.00) | 4.00 (3.00-6.00) | 0.143 |
| **Cardiovascular features** |  |  |  |
| Sinus diameter (mm) | 41.72±15.07 | 47.31±15.06 | 0.122 |
| Ascending aorta diameter (mm) | 41.14±18.89 | 44.74±15.87 | 0.487 |
| Descending aorta diameter (mm) | 30.00±3.46 | 28.43±5.03 | 0.640 |
| Hypertension | 2 (3.57%) | 2 (2.17%) | 0.611 |
| Aortic insufficiency |  |  | 0.184 |
| none | 43 (76.79%) | 60 (65.22%) |  |
| mild | 5 (8.93%) | 19 (20.65%) |  |
| moderate | 2 (3.57%) | 1 (1.09%) |  |
| severe | 6 (10.71%) | 12 (13.04%) |  |
| Mitral regurgitation |  |  | 0.969 |
| none | 42 (75.00%) | 66 (71.74%) |  |
| mild | 11 (19.64%) | 21 (22.83%) |  |
| moderate | 1 (1.79%) | 2 (2.17%) |  |
| severe | 2 (3.57%) | 3 (3.26%) |  |
| Mitral valve prolapse | 5 (8.93%) | 7 (7.61%) | 0.775 |
| **Ocular features** |  |  |  |
| Lens dislocation | 4 (7.14%) | 29 (31.52%) | <0.001 |
| Myopia | 30 (53.57%) | 54 (58.70%) | 0.542 |
| **Skeletal features** |  |  |  |
| Skeletal deformity | 42 (75.00%) | 57 (61.96%) | 0.102 |
| Wrist sign | 41 (73.21%) | 54 (58.70%) | 0.074 |
| Finger sign | 38 (67.86%) | 52 (56.52%) | 0.171 |
| Wrist and Finger sign | 39 (69.64%) | 54 (58.70%) | 0.181 |
| Thoracic deformity | 31 (55.36%) | 26 (28.26%) | 0.001 |
| Scoliosis | 13 (23.21%) | 17 (18.48%) | 0.487 |
| Lung | 6 (10.71%) | 5 (5.43%) | 0.235 |
| Hernia | 3 (5.36%) | 7 (7.61%) | 0.597 |

## Supplementary table 3: The frequency of aortic dissection is higher in the Haploinsufficiency group.

| **Variables** | **Haploinsufficiency** | **Dominant negative** | ***P*-value** |
| --- | --- | --- | --- |
| **Diagnosis** |  |  |  |
| Aneurysm | 5 (19.23%) | 33 (68.75%) | <0.001 |
| Dissection | 21 (80.77%) | 15 (31.25%) | 0.004 |

**Supplementary table 4: The frequencies of frameshift mutations and Haploinsu -fficiency were higher in the aortic dissection group.**

| **Variables** | **Aneurysm (N=46)** | **Dissection (N=43)** | ***P*-value** |
| --- | --- | --- | --- |
| **Type of mutation** |  |  |  |
| missense mutation | 32 (69.57%) | 14 (32.56%) | <0.001 |
| splicing mutation | 8 (17.39%) | 7 (16.28%) | 0.889 |
| frameshift mutation | 2 (4.35%) | 11 (25.58%) | 0.005 |
| nonsense mutation | 4 (8.70%) | 11 (25.58%) | 0.033 |
| **Mutation classification** |  |  |  |
| Haploinsufficiency | 5 (13.16%) | 21 (58.33%) | <0.001 |
| Dominant negative | 33 (86.84%) | 15 (41.67%) | <0.001 |
