# Supplementary material for: Increased frequency of FBN1 frameshift and nonsense mutations in Marfan syndrome patients with aortic dissection
Source: Mol Genet Genomic Med. 2019 Dec 12;8(1):e1041. doi: 10.1002/mgg3.1041 (PMC6978253; doi:10.1002/mgg3.1041)
Supplement: Supplementary file 2 [file MGG3-8-e1041-s002.doc]

## Supplementary table 2: Characteristics between Haploinsufficiency and Dominant negative are the same.

| **Variables** | **Haploinsufficiency(N=56)** | **Dominant negative (N=92)** | ***P*-value** |
| --- | --- | --- | --- |
| **Basic features** |  |  |  |
| Age of onset (year) | 27.00 (10.50-32.00) | 27.00 (19.00-35.00) | 0.233 |
| Body mass index (kg/m^2^) | 20.51±3.91 | 19.72±4.67 | 0.310 |
| Gender |  |  | 0.367 |
| femal | 28 (50.00%) | 39 (42.39%) |  |
| male | 28 (50.00%) | 53 (57.61%) |  |
| Family history | 33 (58.93%) | 54 (58.70%) | 0.978 |
| System score | 5.00 (3.75-7.00) | 4.00 (3.00-6.00) | 0.143 |
| **Cardiovascular features** |  |  |  |
| Sinus diameter (mm) | 41.72±15.07 | 47.31±15.06 | 0.122 |
| Ascending aorta diameter (mm) | 41.14±18.89 | 44.74±15.87 | 0.487 |
| Descending aorta diameter (mm) | 30.00±3.46 | 28.43±5.03 | 0.640 |
| Hypertension | 2 (3.57%) | 2 (2.17%) | 0.611 |
| Aortic insufficiency |  |  | 0.184 |
| none | 43 (76.79%) | 60 (65.22%) |  |
| mild | 5 (8.93%) | 19 (20.65%) |  |
| moderate | 2 (3.57%) | 1 (1.09%) |  |
| severe | 6 (10.71%) | 12 (13.04%) |  |
| Mitral regurgitation |  |  | 0.969 |
| none | 42 (75.00%) | 66 (71.74%) |  |
| mild | 11 (19.64%) | 21 (22.83%) |  |
| moderate | 1 (1.79%) | 2 (2.17%) |  |
| severe | 2 (3.57%) | 3 (3.26%) |  |
| Mitral valve prolapse | 5 (8.93%) | 7 (7.61%) | 0.775 |
| **Ocular features** |  |  |  |
| Lens dislocation | 4 (7.14%) | 29 (31.52%) | <0.001 |
| Myopia | 30 (53.57%) | 54 (58.70%) | 0.542 |
| **Skeletal features** |  |  |  |
| Skeletal deformity | 42 (75.00%) | 57 (61.96%) | 0.102 |
| Wrist sign | 41 (73.21%) | 54 (58.70%) | 0.074 |
| Finger sign | 38 (67.86%) | 52 (56.52%) | 0.171 |
| Wrist and Finger sign | 39 (69.64%) | 54 (58.70%) | 0.181 |
| Thoracic deformity | 31 (55.36%) | 26 (28.26%) | 0.001 |
| Scoliosis | 13 (23.21%) | 17 (18.48%) | 0.487 |
| Lung | 6 (10.71%) | 5 (5.43%) | 0.235 |
| Hernia | 3 (5.36%) | 7 (7.61%) | 0.597 |

## Supplementary table : The frequency of aortic dissection is higher in the Haploinsufficiency group.

| **Variables** | **Haploinsufficiency** | **Dominant negative** | ***P*-value** |
| --- | --- | --- | --- |
| **Diagnosis** |  |  |  |
| Aneurysm | 5 (19.23%) | 33 (68.75%) | <0.001 |
| Dissection | 21 (80.77%) | 15 (31.25%) | 0.004 |

**Supplementary table : The frequencies of frameshift mutations and Haploinsu -fficiency were higher in the aortic dissection group.**

| **Variables** | **Aneurysm (N=46)** | **Dissection (N=43)** | ***P*-value** |
| --- | --- | --- | --- |
| **Type of mutation** |  |  |  |
| missense mutation | 32 (69.57%) | 14 (32.56%) | <0.001 |
| splicing mutation | 8 (17.39%) | 7 (16.28%) | 0.889 |
| frameshift mutation | 2 (4.35%) | 11 (25.58%) | 0.005 |
| nonsense mutation | 4 (8.70%) | 11 (25.58%) | 0.033 |
| **Mutation classification** |  |  |  |
| Haploinsufficiency | 5 (13.16%) | 21 (58.33%) | <0.001 |
| Dominant negative | 33 (86.84%) | 15 (41.67%) | <0.001 |
