# Supplementary material for: Increased frequency of FBN1 frameshift and nonsense mutations in Marfan syndrome patients with aortic dissection
Source: Mol Genet Genomic Med. 2019 Dec 12;8(1):e1041. doi: 10.1002/mgg3.1041 (PMC6978253; doi:10.1002/mgg3.1041)
Supplement: Supplementary file 3 [file MGG3-8-e1041-s003.doc]

**Supplementary table：**A high proportion of aortic dissection in MFS patients with FBN1 nonsense and frameshift mutations

|  | **Missense mutation** | **Splicing mutation** | **Frameshift and nonsense mutation** | ***P-value*** |
| --- | --- | --- | --- | --- |
| **Dissection** | 14 (30.43%) | 7 (46.67%) | 22 (78.57%) | <0.001 |
| **Aneurysm** | 32 (69.57%) | 8 (53.33%) | 6 (21.43%) | <0.001 |
